# Personal NO_2_ and Volatile Organic Compounds Exposure Levels are Associated with Markers of Cardiovascular Risk in Women in the Cape Town Region of South Africa

**DOI:** 10.3390/ijerph16132284

**Published:** 2019-06-28

**Authors:** Frans Everson, Patrick De Boever, Tim S. Nawrot, Nandu Goswami, Mashudu Mthethwa, Ingrid Webster, Dries S. Martens, Nyiko Mashele, Sana Charania, Festus Kamau, Hans Strijdom

**Affiliations:** 1 Division of Medical Physiology, Faculty of Medicine and Health Sciences, Stellenbosch University, P.O. Box 241, Cape Town 8000, South Africa; 2 Health Unit, Flemish Institute for Technological Research (VITO), 2400 Mol, Belgium; 3 Centre for Environmental Sciences, Hasselt University, 3590 Diepenbeek, Belgium; 4 Division of Physiology, Otto Loewi Research Center of Vascular Biology, Immunity and Inflammation, Medical University of Graz, 8036 Graz, Austria

**Keywords:** air pollution, nitrogen dioxide, BTEX, cardiovascular risk, South Africa

## Abstract

Exposure to ambient NO_2_ and benzene, toluene ethyl-benzene and m+p- and o-xylenes (BTEX) is associated with adverse cardiovascular effects, but limited information is available on the effects of personal exposure to these compounds in South African populations. This 6-month follow-up study aims to determine 7-day personal ambient NO_2_ and BTEX exposure levels via compact passive diffusion samplers in female participants from Cape Town, and investigate whether exposure levels are associated with cardiovascular risk markers. Overall, the measured air pollutant exposure levels were lower compared to international standards. NO_2_ was positively associated with systolic and diastolic blood pressure (SBP and DBP), and inversely associated with the central retinal venular equivalent (CRVE) and mean baseline brachial artery diameter. o-xylene was associated with DBP and benzene was strongly associated with carotid intima media thickness (cIMT). Our findings showed that personal air pollution exposure, even at relatively low levels, was associated with several markers of cardiovascular risk in women residing in the Cape Town region.

## 1. Introduction

Ambient air pollution is a global health concern and is associated with numerous adverse health effects including cardiovascular disease (CVD) [1,2,3]. The health effects of ambient air pollution are mostly attributable to small particles and chemically reactive compounds with pro-oxidative potential [4,5,6,7]. Previous reports have suggested that the adverse cardiovascular effects associated with air pollution exposure may be due to autonomic nervous system dysregulation of vascular tone and heart rates [8,9], and pro-atherosclerotic processes such as oxidative stress, inflammation, and endothelial dysfunction [9,10,11]. Although several chemical components present in ambient air have been implicated in adverse cardiovascular outcomes (e.g., nitrogen and carbon oxides, and particulate matter (PM)), the specific contributions and underlying mechanisms of exposure to individual components are not well understood [1,12,13]. 

The World Health Organization (WHO) has identified gaseous pollutants such as NO_2_ and PM with a diameter of ≤2.5 µm (PM_2.5_) as the air pollutants that are most dangerous to public health [14]. Other gaseous pollutants in ambient air include polycyclic aromatic hydrocarbons (PAHs) such as benzene, toluene ethyl-benzene and m+p- and o-xylenes (BTEX) [15,16,17,18]. These gaseous pollutions are mostly produced as a result of the incomplete combustion of fossil fuels during industrial, vehicle, and household activities and are therefore considered a good proxy for general air quality and combustion-related emissions [15,16,17,18]. Due to their small molecular size (molecular diameter: <0.1 nm), these ambient air pollutants are able to passively enter the blood circulation during respiration (predominant route of exposure) and disseminate throughout the body, even at the cellular and nuclear levels where they can exert their harmful effects [1,7,19]. 

Reports on air pollution levels and related health effects are mostly based on data from the developed world, while the situation in developing countries remains under-reported [20,21,22,23]. A recently published systematic review by Katoto et al. (2019) highlighted the lack of air pollution data from the sub-Saharan Africa (SSA) region [23]. The authors identified only 23 published research articles from SSA (of which 14 were from South Africa) that reported on the health effects associated with ambient air pollution. It is evident, however, that data from personal quantitative exposure measurements are under-reported, and none of these studies investigated the effects of air pollution on vascular health and function. The review furthermore reported on studies demonstrating urban air pollution levels of up to 10–20 times greater than recommended WHO standards in some SSA locations [23]. In light of these findings and knowledge gaps, the overarching aims of the current study were to: (1) determine current levels of personal NO_2_ and BTEX exposure during two 1-week time periods in a repeated-measurements study (6-month follow-up) of apparently healthy women residing in the Cape Town region of South Africa, and (2) determine whether current levels of personal NO_2_ and BTEX exposure are associated with markers of cardiovascular risk, including blood pressure (systolic blood pressure (SBP) and diastolic blood pressure (DBP), respectively), flow-mediated vasodilatation (FMD), retinal blood vessel widths, and carotid intima media thickness (cIMT). 

## 2. Materials and Methods

### 2.1. Study Ethics, Design, and Population

The current study formed part of a larger parent study called EndoAfrica and participants for the current study were recruited from the healthy control study group of the parent study [24]. Ethical clearance for the current study was obtained from the Health Research Ethics Committee of Stellenbosch University (ethics reference number: S16/07/114), which subscribes to the principles of the Helsinki Declaration (1975). The study followed a non-interventional, longitudinal (6-month follow-up) cohort design. Healthy volunteering female participants of mixed ancestry were randomLy recruited for the first assessment visit (baseline) from September 2016 to August 2017 at primary health care clinics in the Cape Town region. All participants were from the residential areas of Elsies River, Bishop Lavis, and Ravensmead. All 6-month assessment visits (follow-up) were completed by February 2018. Qualified research nurses recruited, screened, and obtained informed consent from all participants. Participants who were <18 years of age, with human immunodeficiency virus/acquired immunodeficiency syndrome (HIV/AIDS; confirmed with a rapid HIV test; SD Bioline HIV 1/2 3.0 immunochromatographic test kit; Standard Diagnostics, Republic of Korea), with current tuberculosis (confirmed from participant clinic files), pregnant (confirmed with a pregnancy test), <3 months post-partum, of poor health, or with a current or previous history of heart disease were excluded. 

### 2.2. Air Quality and Temperature Monitoring

**Figure A1:**
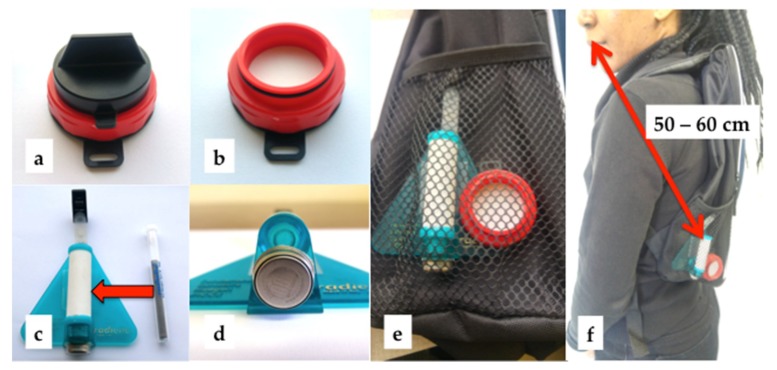
Photos of samplers used for personal exposure measurements: (**a**) Gradko rapid air NO_2_ sampler (product: DIFRAM-100; UKAS method: GLM 7; detection limit: <0.2 µg/m^3^ for 1-week exposure); (**b**) activated Gradko rapid air NO_2_ sampler; (**c**) Radiello^TM^ diffusive cartridge (right) and diffusive body attached to a triangular support plate (left) (products: Rad130, RAD120, and RAD121; detection limit for BTEX: 0.05, 0.01, 0.01, and 0.01 µg/m^3^, respectively; calibration: CS_2_); (**d**) ACR Systems Inc. temperature logger (product: SmartButton (01-0187); detection limit: −40 °C to 85 °C); (**e**) air monitoring devices in the mesh pocket of a backpack (product: Barron BB0110 Curve and Arch Design backpacks); (**f**) backpack worn by participants with samplers located 50 to 60 cm from the face.

Each participant was equipped with a Gradko rapid NO_2_ device (Gradko International Ltd., Winchester, UK), a Radiello^TM^ BTEX passive diffusion sampler (Sigma-Aldrich Inc., MO, USA), and an ACR SmartButton^®^ temperature logger (ACR Systems Inc., Surrey, B.C., Canada) placed in the external mesh pocket of a backpack (Figure A1a–d). The mesh pocket allowed for unrestricted continuous contact with ambient indoor and outdoor temperature and air (Figure A1e). Although previous studies have measured personal sampling within a 30 cm hemisphere from the face [25,26], personal exposure for the current study was measured within a hemisphere of 50 to 60 cm from the face (Figure A1f). Participants carried the backpack at all times (except during periods of sleep and bathroom use when the backpack was placed next to their beds) for a 7-day period. Temperature was recorded continuously at 30-minute intervals while the NO_2_ and BTEX samplers allowed for continuous passive diffusion and accumulation of NO_2_ and BTEX. Following continuous 7-day measurements, participants returned for the first assessment visit, during which the backpacks were collected, data were extracted from the temperature loggers via ACR TrendReader^®^ software (ACR Systems Inc., Surrey, B.C., Canada), and the average temperature (°C) for the 7-day period was recorded. Once collected, the NO_2_ and BTEX samplers were sealed, stored at 4 °C and sent for quantification according to the manufacturer’s protocol. 

NO_2_ samplers were sent to Gradko International Ltd. laboratories (United Kingdom Accreditation Services (UKAS) accredited)) for quantification according to UKAS method GLM 7 [27]. A calibration curve (blank with only deionized water, 15, 30, 60, 90 120 µg/mL) was prepared from a standard nitrate solution (1 g/L nitrite ion (NO_2_^−^)). The color reagent was prepared as previously described [28] and used in a sample:sulphanilamide solution:N-1 (naphthyl-1) ethylene diamine dihydrochloride solution (NEDD) ratio of 1:2:2 (0.003 g NEDD per 1 g sulphanilamide). After sample elution (preparation: 20 % triethanolamine (TEA) solution/80% deionized water), NO_2_ concentrations were determined via chemiluminescence ultraviolet (UV) spectrophotometry (UVS04 Camspec M550; Spectronic Camspec Ltd., Leeds, UK). Calibration standards and linearity checks were used to calibrate the spectrophotometer, and mid-range and zero standards were analyzed at intervals throughout the sequence for quality assurance. The calibration curve was used to calculate the NO_2_^-^ concentration for each sample. The ambient NO_2_ concentration was calculated from NO_2_^−^ concentrations and expressed as µg/m^3^ [29]. 

BTEX samplers were sent to the Flemish Institute for Technology and Research (VITO; Mol, Belgium) for quantification as previously described [30,31]. BTEX samples were extracted from exposed samplers by means of elution in the Radiello^TM^ glass tube containing the cartridge (2 mL carbon disulphide (CS_2_; Sigma-Aldrich, MO, USA) and 12.5 µL 2-fluorotoluene internal standard (Sigma-Aldrich, MO, USA)). The tube was stirred for 30 minutes on a rotational shaker. BTEX quantification was performed by means of gas chromatography (Thermo Trace) and a mass spectrometer (Thermo DSQ II with helium as carries gas (a constant flow of 1 mL/min)). A cross-bond diphenyl/dimethylpolysiloxane column (RTX 502.2; 0.25 mm by 30 m) with a 1.4 µm-thick film was used for sample separation (temperature program: 35 °C for 5 minutes, 14 °C/minute increment until 245 °C). Equipment calibration was performed by injecting the standard solution (benzene, ethyl-benzene, toluene, m-xylene, p-xylene, and o-xylene in CS_2_ (Sigma-Aldrich, MO, USA)) at 0.03 to 30 µg/g before analysis. Sample concentrations were calculated from chromatograms using a standard curve. The limit of detection (LOD) was calculated as 3.3 (standard deviation (SD) of areas/slope). Samples with conc. <LOD were not presented. Results were expressed as average NO_2_ and BTEX exposure concentration (μg/m^3^) for the 7-day measuring period. The same procedure was followed for the 6-month follow-up visits.

### 2.3. Study Endpoints

#### 2.3.1. Health Questionnaire and Anthropometric Measurements

At each visit, participants completed a comprehensive questionnaire to collect data on age, smoking status (defined as smoking or non-smoking), socioeconomic information (including employment status defined as either unemployed, part-time employed, or full-time employed), and lifestyle (hours of sleep at night defined as 1 to 3 hours, between 3 and 6 hours, 6 to 9 hours, and >9 hours). Qualified research nurses measured fasting blood glucose levels (mmol/L) by finger prick (Gluco Plus^TM^, Cipla DIBCARE, Bellville, South Africa) and collected fasting blood and mid-stream urine samples. Cardiovascular measurements included SBP and DBP (calculated as an average of 3 measurements at 5-minute intervals on the left arm and expressed in mmHg) via an Omron M6 automatic digital blood pressure monitor (Omron Healthcare, Kyoto, Japan). Hypertension was defined as either SBP of ≥140 mmHg or DBP of ≥90 mmHg, based on the South African Hypertension Society guidelines [32]. Body mass (kg) was determined on an electronic scale, and height (cm) was decided by means of a stadiometer. Finally, body-mass index (BMI) was expressed as kg body weight/m^2^ height.

#### 2.3.2. Biochemical Analysis

Fasting whole blood was collected in blood collection tubes (SGVac, The Scientific Group (Pty) Ltd.; Milnerton, Western Cape, SA). Serum was extracted from whole blood samples and stored at −80 °C. Serum samples were analyzed by the South African National Health Laboratory Service (NHLS; Tygerberg Hospital, Cape Town, South Africa), a South African National Accreditation System (SANAS)-accredited laboratory, for high-sensitivity C-reactive protein (hsCRP) levels by means of an IMMAGE^®^ Immunochemistry Systems and Calibrator 5 Plus assay kit (Beckman Coulter, Inc., CA, USA). This specific chemiluminescence analysis was based on the highly sensitive Near Infrared Particle Immunoassay rate methodology where anti-CRP antibody-coated particles bind to the CRP in the serum sample resulting in an insoluble aggregate formation and turbidity. Samples were prepared for analysis by adding 4.5 µL serum sample, 42 µL antibody-coated particles (particle-bound goat and mouse anti-CRP antibody), 125 µL buffer 4 and 42 µL diluent (sodium azide: <0.1% (*w*/*w*)). The hsCRP concentration was determined automatically as the rate of aggregate formation (directly proportional). For the purposes of this study, increased hsCRP was defined as hsCRP levels of >3 mg/L based on previous reports suggesting that hsCRP levels above this cut-off value are associated with increased cardiovascular risk [33]. 

Mid-stream urine samples were obtained at each visit, placed on ice and immediately delivered to the NHLS for the quantification of urinary creatinine levels by means of chemiluminescence (cobas^®^-c analyzer and CREP2 kit; Roche Diagnostics, Basel, Switzerland). The principal of the method was based on the enzymatic formation of hydrogen peroxide (catalyzed by peroxidase) that reacted with 2,4,6-triiodo-3-hydroxybenzoic acid for quinone imine chromogen. Creatinine levels were determined by the color intensity of the quinone imine chromogen (directly proportional).

#### 2.3.3. Urinary Analysis of Metabolites of Volatile Organic Compounds

Additional mid-stream urine samples were stored at −80 °C and sent to VITO (Mol, Belgium) for level determinations of the following urinary metabolites: N-acetyl-S-(3-hydroxypropyl)-L-cysteine (HPMA; a marker of acrolein exposure [34]), N-acetyl-s-(phenyl)-L-cysteine (PMA; a marker of benzene exposure [35]), N-acetyl-s-(benzyl)-L-cysteine (BMA; a marker of toluene exposure [36]), trans,trans-muconic acid (MU; a marker of benzene exposure [35]), and 3+4-methylhippuric acid (3+4MHA; a marker of o-, m-, and p-xylene exposure [37]). Samples were prepared using 10 µL urine, 25 µL mixed internal standard (2000 ng/mL in methanol:water(1:1, *v*:*v*)) MU-d4 and 3 (Santa Cruz Biotechnology, TX, USA), and 4 MHA-d7 (Toronto research chemicals Inc., ON, Canada) with 465 µL 1% acetic acid (HAc; Merck, NJ, USA). A matrix-matched calibration curve was applied for the quantification of HPMA, BMA, and PMA to compensate for the matrix effect. To achieve this, spiked urine samples were used containing 10 µL urine, 25 µL mixed internal standard (MU-d4 and 2,3 and 4 MHA-d7: 2000 ng/mL, 20 µL low and high spiked standards (low spike: HPMA, 37.5 ng/mL; PMA, 0.25 ng/mL; MU 5.0 ng/mL, BMA, 1.25 ng/mL; 3+4MHA, 20.0 ng/mL; and high spike: HPMA, 75.0 ng/mL PMA, 0.5 ng/mL; MU, 10.0 ng/mL; BMA, 2.5 ng/mL; 3+4MHA, 40.0 ng/mL (Toronto research chemicals Inc., ON, Canada)) and 445 µL 1% HAc in ultra-pure water. 

Twenty microliters of each sample was injected in an ultra-performance liquid chromatography (UPLS; Waters I-class Acquity UPLC system, Milford, MA, USA)/mass spectrometry (MS; Waters Xevo TQ-S tandem in the negative electrospray ionization mode (ESI^−^)). An Acquity UPLC^®^ high-strength silica T3 column (50 mm × 2.1 mm; 1.8 µm; at a constant temperature of 40 °C) with UV detection (Photodiode array (PDA) detector set at 259 nm) was used for the simultaneous quantification of the urinary metabolites [38]. Retained compounds were eluted with 4 mL HAc solution (10%, *v*:*v*). Levels of metabolites were calculated based on the corresponding matrix-matched calibration curve.

#### 2.3.4. Assessment of Endothelial Function, Carotid Intima Media Thickness, and Retinal Microvascular Caliber 

Vascular endothelial function was assessed via FMD of the right brachial artery, 3–4 cm proximal to the elbow. FMD was measured in the supine position with a mobile Esaote MyLab^TM^ Five portable ultrasound device (Genoa, Italy) with an Esaote Doppler probe (LA523, 12 MHz) connected to computerized software with edge detection technology (Quipu Cardiovascular Suite™; Pisa, Italy) as previously described [24,39]. Briefly, the computerized software determined the mean baseline brachial artery lumen diameter (mm) over a 60-second period, followed by a 5-minute ischaemic occlusion (inflation of a manual blood pressure cuff on the forearm to 50 mmHg supra-systolic pressure). Following the 5-minute ischaemic occlusion, deflating the blood pressure cuff triggered reactive hyperaemia and the maximum brachial artery lumen diameter (µm) was recorded during this period. The maximum lumen diameter displacement during reactive hyperaemia from the mean baseline measurements was expressed as the percentage of the mean baseline brachial lumen diameter (% FMD). 

The cIMTs of the left and right carotid arteries were determined by B-mode ultrasonography as previously described [40,41]. cIMT measurements were performed in the supine position with the head tilted in a 45° angle upwards. The diameter of carotid intima was determined using an Esaote MyLab^TM^ Five portable ultrasound device (Genova, Italy) and a B-linear-mode Esaote Doppler probe (LA523, 12 MHz, Genoa, Italy) connected to computerized software (RF-QIMT software, Genova, Italy) specific for the determination of carotid metrics. Measurements were taken 5 mm proximal to the dilation of the carotid bulb. The mean of the left and right carotid diameter and mean of the left and right cIMT were calculated and used for statistical analysis.

Additionally, retinal images were captured with a Canon CR2 digital camera (Canon Europa NV, The Netherlands) and analyzed with semi-automated software (MONA REVA 2.1.1 developed at VITO; https://mona.health) by trained investigators using a standardized protocol as previously described [24]. Briefly, the central retinal arteriolar equivalent (CRAE) of the 6 largest arterioles and the central retinal venular equivalent (CRVE) of the 6 largest venules were determined in the area between 0.5 and 1 disc diameter from the optic disc margin and also expressed as a CRAE/CRVE ratio (AVR). The calculations of the vessel metrics were based on the revised Parr-Hubbard formulas as reported previously [42].

### 2.4. Statistical Analysis 

All statistical analyses were performed with IBM^®^ SPSS^®^ software (version 25; New York, NY, USA). Depending on data distribution, a paired sample Student’s *t*-test (parametric) or Wilcoxon signed-rank test (non-parametric) was used to identify significant differences between baseline and follow-up visits. A Spearman’s Rho correlation (nonparametric data) was used to identify correlations between variables. To determine the association between NO_2_ or individual BTEX compounds or total BTEX (a summation of all individual BTEX compounds for each participant as a proxy for combined effect of BTEX) and different cardiovascular outcomes, a linear mixed model (LMM) regression analysis was used with participants nested in each visit. Variables with skewed data distribution (BTEX and total BTEX) were log-transformed for regression analysis. To evaluate personal air pollution effects on cardiovascular outcomes independent of potential confounding effects, we selected a priori covariates that are known determinants for cardiovascular outcomes and variables with a potential link with personal air pollution exposure and cardiovascular outcomes. These include age, BMI, smoking, socio-economic status (reflected by employment), sleep, and ambient temperature [43,44,45,46,47]. For estimated effects on SBP and DBP, the statistical model included NO_2_ or BTEX or total BTEX as an exposure variable, participants at each time point as a random effects factor variable with random intercept to account for possible inter-individual variation while adjusting for age, BMI, date of assessment visit, and average temperature as continuous fixed effects and smoking status, employment status, and hours of sleep at night as fixed categorical variables. For effects on other vascular outcomes, we additionally adjusted LMMs for SBP. To determine estimated effects on % FMD and cIMT, the mean brachial diameter and carotid diameter were additionally adjusted for, respectively. Q-Q plots of the residuals were used to test the assumptions of linearity. The significance threshold was set at *p* < 0.05 for all statistical analysis.

## 3. Results

### 3.1. Baseline Population Characteristics

A total number of 77 female participants were recruited for the study. Sixteen participants who completed their baseline visits did not consent to continue with the 6-month follow-up visit and were excluded from the study. A total number of 61 healthy female participants (mean ± SD age at baseline: 42.5 ± 13.4 years) of mixed ancestry completed both assessment visits (Table 1). 

The majority of participants were current smokers (69%; smoking frequency, <20 cigarettes/day), but none reported any history of heart or other current serious health problems. Participants were mostly unemployed (49%). Most participants were overweight with a mean ± SD BMI of 27.7 ± 8.4 kg/m^2^. Most participants (*n* = 37; 61%) reported that they sleep 6 to 9 hours per night. The mean ± SD SBP and DBP values (SBP: 122.5 ± 19.9) mmHg, and DBP: 84.2 ± 12.0 mmHg) were within the normal range (Table 2). In total, 11 participants (18%) presented with hypertension (either SBP of ≥140 mmHg or DBP of ≥90 mmHg) at baseline. The median hsCRP level was above the 3 mg/L cut-off value, and the majority of participants (*n* = 35; 61%) exhibited elevated hsCRP levels. 

### 3.2. Personal Ambient Exposure Variable Outcomes and Urinary Metabolites

The mean NO_2_ and median benzene, ethyl-benzene, m+p-xylene, and o-xylene levels were significantly higher at the baseline visit compared to at the follow-up visit (Table 3). Personal toluene exposure accounted for ~50% of the total BTEX exposure level at baseline and follow-up visits, while benzene exposure was the lowest at both baseline and follow-up visits. The mean temperature did not differ between baseline and follow-up (Table 3). Strong positive correlations were observed between all personal exposure concentrations (*p* < 0.001) (Table A1). No significant differences in urinary metabolites were observed between baseline and follow-up visits. 

### 3.3. Air Pollution Exposure and Cardiovascular Endpoints 

NO_2_ exposure was positively associated with blood pressure and negatively associated with blood vessel diameters. Each SD increment (4.96 µg/m^3^) in NO_2_ was associated with 2.42 mmHg (95% CI: 0.03 to 4.80 mmHg; *p* = 0.047) and 1.76 mmHg (95% CI: 0.00 to 3.52 mmHg; *p* = 0.050) increase in SBP and DBP, respectively (Table 4). Each NO_2_ SD increment was associated with −2.08 µm (95% CI: −4.13 to −0.02 µm; *p* = 0.048) decrease in CRVE and −0.11 mm (95% CI: −0.19 to −0.03 mm; *p* < 0.010) decrease in mean baseline brachial diameter.

BTEX exposure was positively associated with blood pressure and cIMT measurements. Each SD increments in total BTEX (2.56 µg/m^3^) and o-xylene (2.51 µg/m^3^) were associated with a 2.07 mmHg (95% CI: 0.06 to 4.07 mmHg; *p* = 0.043) and 2.00 mmHg (95% CI: 0.21 to 3.80 mmHg; *p* = 0.029) increase in DBP, respectively (Table 4; and Table A2). Each SD (2.08 µg/m^3^) increment increase in benzene was positively associated with 24.88 µm (95% CI: 2.19 to 47.57 µm); *p* = 0.032) increase in cIMT (Table A5). 

The urinary metabolite 3+4MHA was negatively associated with vascular function as indicated by % FMD with an estimated effect of −1.45% (95% CI: −2.38% to −0.51%; *p* = 0.003) for each SD (3.12 ng/mL) increment in 3+4MHA (Table A4). For more detailed results on effects of exposure on vascular outcomes, refer to Table A2, Table A3, Table A4 and Table A5.

## 4. Discussion

The current study set out to determine personal air pollution exposure levels as measured by NO_2_ and BTEX, in a panel study of female adults residing in the Cape Town region of South Africa, and investigated whether these exposure levels are associated with markers of cardiovascular risk. Major findings of the study are: (1) compared to international air quality standards, our participants’ personal exposure to NO_2_ and BTEX was relatively low, and (2) despite the relatively low exposure levels, we could demonstrate associations between air pollutants and several cardiovascular parameters: SBP and DBP (NO_2_ and BTEX), baseline brachial artery diameter (NO_2_), CRVE (NO_2_), and cIMT (benzene). 

The mean 7-day personal NO_2_ exposure concentrations (Table 3) observed during the course of the study (range: 2.94 µg/m^3^–25.35 µg/m^3^) remained below the recommended WHO, European Union (EU) and South African air quality standards for NO_2_: annual exposure of <40 µg/m^3^ and 1-hour exposure of <200 µg/m^3^ [48,49,50,51]. At baseline, 24 (22%) of the individual mean benzene measurements (range: 0.475 µg/m^3^–14.17 µg/m^3^) were higher than WHO and EU annual recommended standards (<5 µg/m^3^), although the median values were below the standards [17,48]. 

Furthermore, the mean NO_2_ and median benzene concentrations at baseline in our study population (NO_2_: 13.6 ± 4.8 µg/m^3^ and benzene: 3.22 (0.77–14.17) µg/m^3^) were within the lower range of annual mean ambient NO_2_ and benzene concentrations of major European cities, with NO_2_ ranging from 8 µg/m^3^ in Stockholm to 43 µg/m^3^ in Barcelona, and benzene ranging between 2 and 12 µg/m^3^ across various cities [3,52,53,54]. 

Compared to other studies from the Western Cape Province, the observed mean personal NO_2_ levels in our study were comparable with recently reported annual mean NO_2_ levels from air quality monitoring stations in neighboring areas (Goodwood: 21 µg/m^3^; Plattekloof: 10 µg/m^3^) [49]. The personal NO_2_ and individual BTEX concentrations in the current study were furthermore similar to those measured previously (2011–2014) in the Drakenstein sub-district, ~60 km from the City of Cape Town [55]. In this study, household NO_2_ and BTEX levels (reported as 2-week median values) were measured in more than 500 homes of families of African and mixed ancestry (NO_2_: 7.9 (Interquartile range (IQR): 3.8–13.3) µg/m^3^, benzene: 5.6 (IQR: 2.6–17.1) µg/m^3^, toluene: 19.8 (IQR: 9.3–53.2) µg/m^3^, ethyl-benzene: 2.1 (IQR: 0.9–53.2) µg/m^3^, m+p-xylene: 5.8 (2.4–16.2) µg/m^3^, and o-xylene 2.4 (IQR: 1.1–7.0) µg/m^3^).

Ambient outdoor BTEX levels are infrequently measured in the Western Cape Province of South Africa. The Provincial Government Report (2013) reported on BTEX levels (measured with passive samplers) from only two locations in one rural town (Riversdale) in the province [49]. The reported values from Riversdale (two locations with benzene concentrations of 0.89 and 1.05 µg/m^3^, respectively, toluene concentrations of 2.66 and 1.04 µg/m^3^, respectively, ethyl-benzene concentrations of 0.43 and 0.27 µg/m^3^, respectively, and xylene concentrations of 1.82 and 1.19 µg/m^3^, respectively,) were generally lower than the personal BTEX exposure levels measured in our study [49].

Our results showed that personal NO_2_ and total BTEX (mostly driven by o-xylene) exposure was positively associated with blood pressure outcomes after adjusting for covariates (Table 4). Both long- and short-term exposure to ambient gaseous pollutants has previously been associated with haemodynamic changes including blood pressure, even at low exposure concentrations [18,56,57,58,59]. 

Our findings support those of Chan et al. (2015) who examined the effects of NO_2_ and fine PM (≤2.5 μm; PM_2.5_) in a female population and showed that a 10 ppb increase in NO_2_ was associated with a higher pulse pressure (0.4 mmHg) [60]. In the same study, PM_2.5_ was also associated with higher SBP (1.4 mmHg), pulse pressure (1.0 mmHg), and mean arterial pressure (0.8 mmHg) [60]. The authors speculated that exposure-associated autonomic dysregulation of vascular tone may be a possible underlying mechanism of their findings [60,61]. This may indeed be the case, as our results showed negative associations between NO_2_ and vessel diameters as indicated by the baseline brachial artery diameter and CRVE. The vasoconstrictive effects of both long-term low-concentrations and short-term high-concentration exposure to ambient air pollution have previously been described [62,63]. In the study by Brook et al. (2002), an inverse relationship with brachial artery diameter (ultrasonography) was also demonstrated in 25 healthy adults, although at higher exposure concentrations (150 µg/m^3^ fine PM and 120 ppb O_3_) and a shorter exposure period (2 hour) compared to our exposure concentrations and exposure period [62]. The authors, in a finding similar to ours, failed to demonstrate ambient air pollution exposure-associated effects on FMD [62].

Previous studies have investigated the possible mechanism involved in air pollution exposure-associated vasoconstriction and suggested possible stimulation of the pulmonary vagal afferent neurons and the subsequent increase in sympathetic nervous system reflex activity or an upregulation (directly or via oxidative stress pathways) of vascular endothelin 1 and 3 (vasoconstrictors) [62,64]; however, more studies are required to elucidate the underlying mechanisms. 

Benzene exposure was associated with sub-clinical atherosclerotic changes as measured by cIMT in our study with a relatively large estimated effect on cIMT. cIMT is considered a marker of pro-atherosclerotic processes such as inflammation, a strong predictor of future cardiovascular events [65], and increased cIMT has previously been implicated in long-term ambient PM exposure [19,66]. It has also been suggested that women are at higher risk of increased PM-induced cIMT than men [19]. Short-term air pollutant exposure levels in our study were not significantly associated with inflammation (as measured by hsCRP), suggesting that other possible mechanisms may explain the pro-atherosclerotic effects of benzene.

3+4MHA appeared to be a prominent urinary metabolite/tracer for exposure in our study as it strongly correlated with all personal exposure levels (Table A1). 3+4MHA is a urinary marker for toluene (80% inhaled toluene metabolized to BMA or MHA) and the primary metabolite for xylene exposure (95% inhaled xylene metabolized to MHA post-exposure) [38,67,68,69,70]. Aside from being a diesel exhaust derivative, xylene is also often used as a solvent in industrial and household products (e.g., adhesives, coatings, degreasers, detergents, dyes, ink, paint, pesticides, polishes, and solvents) [18,56,57,58,59]. 

Xylene is a carcinogen and also associated with central nervous system abnormities such as brain and neurobehavioral morbidities [71,72]. In our study, the positive association between personal total BTEX and DBP was mostly driven by o-xylene, while no adverse associations between BMA (marker of toluene exposure) and vascular endpoints were observed. The significant association between o-xylene and DBP, as well as between its primary urinary metabolite, 3+4MHA (Table A4), and endothelial function suggests that o-xylene may significantly contribute to vascular dysfunction (increased DBP and reduced endothelial function as measured by % FMD) in our study population. More focused investigations on possible underlying mechanisms are required. 

## 5. Strengths and Limitations

The results from the current study are presented with some strengths and limitations. Strengths of current study include the measurement of personal exposure levels, as opposed to levels obtained from centralized air quality monitoring stations in many previous studies from the SSA region. Personal measurements are generally considered a more accurate representation of exposure levels. An additional strength of the study is the fact that we used measurements of different cardiovascular endpoints to investigate physiological effects. To the best of our knowledge, this study is the first to explore the cardiovascular health effects of personal air pollution exposure, in combination with urinary exposure markers, in the South African research setting.

Limitations of the study include a relatively small sample size representing only women, of whom the majority were smokers. The male participant enrolment rate was low, mostly due to employment obligations that resulted in difficulty to attend assessment visits. The high prevalence of smokers in our cohort may be ascribed to the high smoking rates that have previously been reported in the region [73,74]. The robust correction for the effects of smoking on various outcomes, based only on smoking status, may not have been optimal. Using levels of biomarkers of smoking such as cotinine may have been a more accurate adjustment for the smoking effect and would have also included the effects of possible second-hand smoke. Personal exposure measurements with a 30 cm radius extended in front of the face (within the breathing zone) and the inclusion of blank field samples in the study would also have been more desirable. These factors should be considered in future studies. Additionally, as previously shown, the effects of air pollution vary across sex, ethnicity, and health status [75,76,77]. Our results represent only the exposure effects in an apparently healthy, female population of mixed ancestry and care should be taken to not extrapolate our findings to the general population. 

## 6. Conclusions

Our results show that personal ambient air pollution exposure in women residing in Cape Town, even at relatively low levels, is associated with markers of cardiovascular risk including blood pressure (SBP and DBP), vascular tone/diameter (baseline brachial artery diameter and CRVE), vascular endothelial function (% FMD), and subclinical atherosclerosis (cIMT).

## Figures and Tables

**Table 1:** Baseline study population characteristics (*n* = 61).

Variable	Baseline
Age (years)	42.5 ± 13.4
Smoking status	
Current smoker (*n*)	42 (69%)
Employment	
Unemployed (*n*)	30 (49%)
Part-time (*n*)	25 (41%)
Full-time (*n*)	6 (10%)
Hours of sleep per night	
<3 h (*n*)	1 (2%)
3 to ≤6 h (*n*)	7 (12%)
6 to ≤9 h (*n*)	37 (61%)
>9 h (*n*)	16 (25%)
BMI, kg/m^3^ (*n*)	27.7 ± 8.4
High-sensitivity C-reactive protein (mg/L) ^a, b^	6.3 (0.2 to 37.1)
Elevated hsCRP (>3 mg/L)	
Yes (*n*)	35 (61%)
No (*n*)	22 (39%)
Urine creatinine (mmol/L)	13.5 ± 7.1

Data presented as mean ± SD or *n* (%); ^a^ data presented as median (range); ^b^ sample size: *n* = 57.

**Table 2:** Baseline cardiovascular parameters (*n* = 61).

Variable	Baseline
Blood pressure	
Systolic blood pressure (SBP); mmHg)	122.5 ± 19.9
Diastolic blood pressure (DBP; mmHg)	84.15 ± 12.0
Hypertension (Either SBP of >140 mmHg or DBP of >90 mmHg)	
Yes, *n*	15 (25%)
No, *n*	46 (75%)
Flow-mediated vasodilatation ^a^	
Brachial diameter (mm)	3.22 ± 0.69
% Flow-mediated Dilatation (% FMD) ^b^	5.21 (−7.93 to 23.50)
Retinal caliber ^c^	
Central retinal arteriolar equivalent (CRAE; μm)	157.9 ± 16.4
Central retinal venular equivalent (CRVE; μm)	238.4 ± 20.1
CRAE/CRVE ratio (AVR)	0.66 ± 0.06
Carotid artery	
Carotid diameter (mm)	7.16 ± 0.84
Carotid intima media thickness (cIMT; μm)	657.2 ± 159.3

Data presented as mean ± SD or *n* (%); ^a^ sample size: *n* = 60; ^b^ data presented as median (range); ^c^ sample size: *n* = 58.

**Table 3:** Air pollution characteristics at baseline and 6-month follow-up (*n* = 61).

Variable	Baseline	Follow-Up
Temperature ^a, b^ (°C)	21.6 ± 3.2	21.9 ± 2.7
Personal air pollution measurements		
NO_2_^a, c^ (µg/m^3^)	13.6 ± 4.8	10.6 ± 4.7 **
Total BTEX ^c,^ ^d^ (µg/m^3^)	43.0 (12.0 to 327.7)	34.31 (7.1 to 405.1)
Benzene ^c^ (µg/m^3^)	3.9 (0.7 to 14.2)	2.2 (0.5 to 9.3) *
Toluene ^c^ (µg/m^3^)	22.1 (5.6 to 189.2)	18.0 (3.7 to 284.1)
Ethyl-benzene ^c^ (µg/m^3^)	2.8 (1.1 to 34.4)	2.3 (0.7 to 21.4) *
m+p-xylene ^c^ (µg/m^3^)	9.2 (3.4 to 117.4)	7.5 (2.0 to 74.8) *
o-xylene ^c^ (µg/m^3^)	3.2 (1.2 to 43.8)	2.7 (0.7 to 24.7) *
Urinary metabolites ^e^		
HPMA ^f^ (ng/mL)	1686 (92 to 12,793)	1812 (120 to 12,613)
PMA ^g^ (ng/mL)	0.05 (0.05 to 0.34)	0.05 (0.05 to 0.34)
MU ^h^ (ng/mL)	62.5 (62.5 to 498.0)	62.5 (62.5 to 595.0)
BMA ^i^ (ng/mL)	14.7 (2.5 to 588.0)	14.3 (2.5 to 699.0)
3+4MHA ^j^ (ng/mL)	1061 (31.8 to 9512.0)	845 (50.0 to 32,078.0)

Data presented as median (range) or ^a^ mean ± SD; ^b^ the mean represents the average 7-day recorded temperatures (30-minute interval temperature recordings) for each participant prior to the assessment visit, *n* = 60. ^c^ Values reflect the mean 7-day average during the 7-day period prior to the assessment visit, *n* = 56 to 61. ^d^ Values represent the mean values of the sum of individual BTEX measurements for each participant as a proxy for total BTEX exposure. ^e^ Values of samples below limit of detection (LOD) were replaced by LOD/2. ^f^ Samples concentrations above LOD (>80 ng/mL; baseline/follow-up: *n* = 61/61). ^g^ Samples concentrations above LOD (>0.09 ng/mL; baseline/follow-up: *n* = 10/6). ^h^ Samples concentrations above LOD (>125 ng/mL; baseline/follow-up: *n* = 14/20). ^i^ Samples concentrations above LOD (>5 ng/mL; baseline/follow-up: *n* = 46/45). ^j^ samples concentrations above LOD (>100 ng/mL; baseline/follow-up: *n* = 60/58). * *p* < 0.05; ** *p* < 0.01.

**Table 4:** Estimated effects of personal NO_2_ and total BTEX on vascular outcomes.

Variable	Exposure Variable	Estimate ^a,b^ (95% CI)	*p*-Values
SBP ^c^ (mmHg)	NO_2_	2.42 (0.03; 4.80)	0.047
	Total BTEX	1.54 (−1.38; 4.46)	0.297
DBP ^c^ (mmHg)	NO_2_	1.76 (0.00; 3.52)	0.050
	Total BTEX	2.07 (0.06; 4.07)	0.043
CRAE ^d^ (µm)	NO_2_	−0.47 (−2.25; 1.31)	0.599
	Total BTEX	−0.70 (−2.88; 1.47)	0.521
CRVE ^d^ (µm)	NO_2_	−2.08 (−4.14; −0.02)	0.048
	Total BTEX	−0.29 (−3.00; 2.39)	0.829
Mean brachial diameter ^d^ (mm)	NO_2_	−0.11 (−0.19; −0.03)	0.005
	Total BTEX	−0.08 (−0.17; 0.01)	0.090
% FMD ^e^	NO_2_	−0.11 (−1.00; 0.77)	0.801
	Total BTEX	0.30 (−0.56; 1.15)	0.492
Carotid Diameter ^d^ (mm)	NO_2_	−0.06 (−0.19; 0.08)	0.393
	Total BTEX	−0.12 (−0.26; 0.02)	0.082
cIMT ^f^ (µm)	NO_2_	1.23 (−23.63; 26.09)	0.921
	Total BTEX	12.76 (−10.55; 36.06)	0.275

^a^ All models adjusted for date of assessment visit, average temperature, age, body-mass index (BMI), smoking, and employment status (random factor: participant). ^b^ Estimates expressed as a difference in cardiovascular endpoint for each SD increment in exposure. ^c^ Additionally adjusted for hours of sleep at night. ^d^ Additionally adjusted for SBP. ^e^ Additionally adjusted for SBP and mean brachial diameter. ^f^ Additionally adjusted for the SBP and carotid diameter.

## Appendix A

**Table A1:** Spearman’s Rho correlations coefficients (*r*) for all personal measures of exposure and urinary metabolites.

	NO_2_	Benzene	Toluene	Ethyl-benzene	m+p-xylene	o-xylene
Personal Exposure ^a^						
Benzene	0.616 ***					
Toluene	0.452 ***	0.461 ***				
Ethyl-benzene	0.622 ***	0.585 ***	0.652 ***			
m+p-xylene	0.642 ***	0.595 ***	0.658 ***	0.983 ***		
o-xylene	0.643 ***	0.589 ***	0.667 ***	0.972 ***	0.989 ***	
Total BTEX ^b^	0.524 ***	0.474 ***	0.951 ***	0.790 ***	0.798 ***	0.808 ***
Urinary metabolites ^c^						
HPMA	0.081	0.186 *	−0.020	−0.002	0.023	0.017
PMA	0.117	0.011	−0.218 *	−0.112	−0.105	−0.123
MU	0.061	0.152	−0.009	−0.749	−0.038	−0.024
BMA	−0.051	−0.070	0.120	−0.023	−0.018	−0.005
3+4MHA	0.273 **	0.341 ***	0.218 *	0.192 *	0.215 *	0.210 *

^a^*n* = 111 to 113; ^b^ values represent the mean values of the sum of individual BTEX measurements for each participant; ^c^ *n* = 111 to 121; * *p* < 0.05; ** *p* < 0.01; *** *p* < 0.001.

**Table A2:** Estimated effects of personal NO_2_, total BTEX, BTEX, and 3+4MHA urinary metabolite on SBP and DBP.

Variable	Exposure Variable	Estimate ^a,b^ (95% CI)	*p*-Values
SBP ^c^ (mmHg)	NO_2_	2.42 (0.03; 4.80)	0.047
	Total BTEX	1.54 (−1.38; 4.46)	0.297
	Benzene	2.51 (−0.21; 5.22)	0.070
	Toluene	0.92 (−2.04; 3.88)	0.539
	Ethyl-benzene	1.44 (−1.15; 4.03)	0.272
	m+p-xylene	1.62 (−1.01; 4.25)	0.224
	o-Xylene	2.12 (−0.50; 4.74)	0.112
	Urinary metabolite ^d^		
	3+4MHA	−0.52 (−3.10; 2.05)	0.687
DBP ^c^ (mmHg)	NO_2_	1.76 (0.00; 3.52)	0.050
	Total BTEX	2.07 (0.06; 4.07)	0.043
	Benzene	1.19 (−0.73; 3.10)	0.220
	Toluene	1.77 (−0.25; 3.80)	0.086
	Ethyl-benzene	1.58 (−0.20; 3.36)	0.080
	*m*+*p*-xylene	1.72 (−0.09; 3.53)	0.062
	o-xylene	2.01 (0.21; 3.80)	0.029
	Urinary metabolite ^d^		
	3+4MHA	−0.40 (−2.28; 1.48)	0.673

^a^ All models adjusted for date of assessment visit, average temperature, age, BMI, smoking, and employment status (random factor: participant). ^b^ Estimates expressed as a difference in cardiovascular endpoint for each SD increment in exposure. ^c^ Additionally adjusted for hours of sleep at night. ^d^ Additionally adjusted for hours of sleep at night and urine creatinine.

**Table A3:** Estimated effects of personal NO_2_, total BTEX, BTEX, and 3+4MHA urinary metabolite on CRAE and CRVE.

Variable	Exposure Variable	Estimate ^a,b^ (95% CI)	*p*-Values
CRAE ^c^ (µm)	NO_2_	−0.47 (−2.25; 1.31)	0.599
	Total BTEX	−0.70 (−2.88; 1.47)	0.521
	Benzene	−0.54 (−2.51; 1.42)	0.582
	Toluene	−0.74 (−2.90; 1.43)	0.500
	Ethyl-benzene	−0.49 (−2.24; 1.26)	0.579
	m+p-xylene	−0.60 (−2.40; 1.21)	0.511
	o-xylene	−0.68 (−2.53; 1.16)	0.461
	Urinary metabolite ^d^		
	3+4MHA	−0.57 (−2.297; 1.167)	0.517
CRVE ^c^ (µm)	NO_2_	−2.08 (−4.14; −0.02)	0.048
	Total BTEX	−0.29 (−3.00; 2.39)	0.829
	Benzene	−0.67 (−3.11; 1.78)	0.588
	Toluene	−0.40 (−3.11; 2.31)	0.768
	Ethyl-benzene	−0.53 (−2.64; 1.58)	0.616
	m+p-xylene	−0.50 (−2.62; 1.62)	0.638
	o-xylene	−0.61 (−2.85; 1.63)	0.587
	Urinary metabolite ^d^		
	3+4MHA	−0.14 (−2.19; 1.91)	0.890

^a^ All models adjusted for date of assessment visit, age, BMI, average temperature, smoking, and employment status (random factor: participant). ^b^ Estimates expressed as a difference in cardiovascular endpoint for each SD increment in exposure. ^c^ Additionally adjusted for SBP. ^d^ Additionally adjusted for SBP and urine creatinine.

**Table A4:** Estimated effects of personal NO_2_, total BTEX, BTEX, and 3+4MHA urinary metabolite on FMD measurements.

Variable	Exposure Variable	Estimate ^a,b^ (95% CI)	*p*-Values
Mean brachial diameter ^c^ (mm)	NO_2_	−0.11 (−0.19; −0.03)	0.005
	Total BTEX	−0.08 (−0.17; 0.01)	0.090
	Benzene	−0.01 (−0.10; 0.08)	0.760
	Toluene	−0.09 (−0.17; 0.01)	0.065
	Ethyl-benzene	−0.08 (−0.17; 0.00)	0.057
	m+p-xylene	−0.06 (−0.15; 0.02)	0.144
	o-xylene	−0.06 (−0.15; 0.03)	0.189
	Urinary metabolite ^d^		
	3+4MHA	0.02 (−0.07; 0.11)	0.647
% FMD ^e^	NO_2_	−0.11 (−1.00; 0.77)	0.801
	Total BTEX	0.30 (−0.56; 1.15)	0.492
	Benzene	−0.01 (−0.87; 0.85)	0.982
	Toluene	0.36 (−0.50; 1.22)	0.403
	Ethyl-benzene	0.35 (−0.76; 0.90)	0.870
	m+p-xylene	0.07 (−0.77; 0.92)	0.862
	o-xylene	0.16 (−0.68; 1.00)	0.705
	Urinary metabolite ^f^		
	3+4MHA	−1.45 (−2.38; −0.51)	0.003

^a^ All models adjusted for date of assessment visit, age, BMI, average temperature, smoking, and employment status (random factor: participant). ^b^ Estimates expressed as a difference in cardiovascular endpoint for each SD increment in exposure. ^c^ Additionally adjusted for SBP. ^d^ Additionally adjusted for SBP and urine creatinine. ^e^ Additionally adjusted for SBP and baseline brachial diameter. ^f^ Additionally adjusted for the SBP, baseline brachial diameter, and urine creatinine.

**Table A5:** Estimated effects of personal NO_2_, total BTEX, BTEX, and 3+4MHA urinary metabolite on carotid artery measurements.

Variable	Exposure Variable	Estimate ^a,b^ (95% CI)	*p*-Values
Carotid diameter ^c^ (mm)	NO_2_	−0.06 (−0.19; 0.08)	0.393
	Total BTEX	−0.12 (−0.26; 0.02)	0.082
	Benzene	−0.07 (−0.20; 0.07)	0.331
	Toluene	−0.11 (−0.25; 0.03)	0.132
	Ethyl-benzene	−0.10 (−0.22; 0.02)	0.097
	m+p-xylene	−0.10 (−0.23; 0.02)	0.109
	o-xylene	−0.09 (−0.22; 0.04)	0.156
	Urinary metabolite ^d^		
	3+4MHA	−0.07 (−0.20; 0.07)	0.325
cIMT ^e^ (µm)	NO_2_	1.23 (−23.63; 26.09)	0.921
	Total BTEX	12.76 (−10.55; 36.06)	0.275
	Benzene	24.88 (2.19; 47.57)	0.032
	Toluene	9.37 (−13.24; 31.99)	0.407
	Ethyl-benzene	9.10 (−14.32; 32.52)	0.437
	m+p-xylene	8.64 (−15.36; 32.64)	0.471
	o-xylene	13.06 (−10.74; 36.85)	0.274
	Urinary metabolite ^f^		
	3+4MHA	−10.56 (−30.75; 9.63)	0.302

^a^ All models adjusted for date of assessment visit, age, BMI, average temperature, smoking, and employment status (random factor: participant). ^b^ Estimates expressed as a difference in cardiovascular endpoint for each SD increment in exposure. ^c^ Additionally adjusted for SBP. ^d^ Additionally adjusted for SBP and urinary creatinine. ^e^ Additionally adjusted for SBP and mean brachial diameter. ^f^ Additionally adjusted for the SBP, mean carotid diameter, and urinary creatinine.
